# Formation of Self-Connected Si_0.8_Ge_0.2_ Lateral Nanowires and Pyramids on Rib-Patterned Si(1 1 10) Substrate

**DOI:** 10.1186/s11671-016-1820-z

**Published:** 2017-01-24

**Authors:** Lei Du, Gang Chen, Wei Lu

**Affiliations:** National Laboratory for Infrared Physics, Shanghai Institute of Technical Physics, Chinese Academy of Sciences, 500 Yutian Road, Shanghai, 200083 China

**Keywords:** Germanium, Lateral nanowires, Finite-element simulation, Patterned template, Molecular beam epitaxy

## Abstract

In this work, Si_0.8_Ge_0.2_ is deposited onto the rib-patterned Si (1 1 10) template oriented in the [1 −1 0] direction. Atomic force microscopy (AFM) reveals that the rib sidewalls reshape into pyramid-covered (0 0 1) and smooth {1 1 3} facets, respectively, while the {1 0 5} facets-bounded lateral SiGe nanowires dominate the rib top along the [5 5 −1] direction. At both the rib shoulder sites and the pyramid vacancy sites, self-connecting occurs between the meeting nanowire and pyramids to form elongated huts, which are driven by the minimization of the total energy density according to the finite-element simulations results. These results suggest a convenient solution to form lateral SiGe nanowires covering multi-faceted surfaces on the patterned template.

## Background

As an alternative candidate for the miniaturized complementary metal oxide semiconductor field effect transistors (MOSFET) [1–3], and many other promising applications [4–6], Ge-based nanowires have been extensively studied owing to their compatibility with the well-established Si-based semiconductor technology. So far, the bottom-up vapor-liquid-solid (VLS) growth method has been successfully developed and applied to fabricate Ge and Ge/Si core/shell nanowires with excellent device performance [4, 7]. Nevertheless, there has not been any convenient solution available to transfer/arrange these vertically grown nanowires yet, which limits its potential application in microelectronics [6]. On the other hand, recently laterally aligned heteroepitaxial in-plane Ge nanowires on Si substrate have been regarded as an alternative solution on both singular [8–12] and miscut [13, 14] Si (0 0 1) and Si (1 1 1) [15, 16] substrates, especially the Si (1 1 10) surface [17–19], where the lateral nanowires can naturally extend along the [5 5 −1] direction. The mechanism behind the formation and stability of these nanowires are attributed to abnormal faceting of the wetting layer [6, 13] or extended huts [10, 20], which is driven by the low surface energy of their {1 0 5} side facets under equilibrium phase. These lateral nanowires have shown potential applications for nano-electronics [10, 21], spintronic devices [22, 23], and optoelectronics [24, 25].

To further explore the excellent features of SiGe lateral nanowires, it becomes necessary to fully understand the mechanism of the nanowire formation on various substrates for the steerable growth. So far, template patterning is one of the most successful routes to achieve controllable growth of the lateral SiGe nanowires on both miscut [19] and singular Si (001) substrate [26]. Recent work has also showed the morphology evolution between the nanowires and the islands during the variation of the growth temperature and the thickness of the Si spacer [27, 28]. Nevertheless, there has been very few reports on the evolution of the SiGe lateral nanowires on the patterned miscut substrate, where the coexistence of the nanowires and islands are inevitable. It is necessary to study the controlled growth of SiGe nanowires on patterned templates, while the interaction between the nanowires and the islands on multi-faceted template, and especially at the shoulders or connecting edges, also has to be investigated.

Therefore, here we apply a different strategy for the template patterning from what have been used before. In our previous work, the ribs are patterned onto the Si (1 1 10) substrate along the [5 5 −1] direction to ensure the formation of micromillimeter-long nanowires via the geometry-induced self-elongation and self-alignment effects [19]. On the other hand, in this work, the ribs are patterned along the [1 −1 0] orientation which is perpendicular to the expected SiGe nanowire direction. Since the SiGe nanowires self-align along the [5 5 −1] direction, such a configuration will truncate the nanowires at the shoulder of the ribs [29]. With a well-tuned lithography technique and the buffer layer growth conditions, the (001) faceted sidewalls are introduced adjacent to the (1 1 10) terminated rib top. Thus, we can study the interactions between the nanowires and other self-assembled nano-structures on two adjacent facets.

As for the choice of the SiGe concentration, we focused on the Si_0.8_Ge_0.2_ nanowires in this work due to the promising features both in thermal transport [30] and carrier transport [31] for Si-rich Si_1-x_Ge_x_ heterostructures (especially when *x* = 0.2), which recently have been intensively explored for potential thermoelectronic applications [32, 33], and for achieving semiconductor quantum dot spin qubit [34–36].

Thus, we studied the behavior of the formation of the nanowires and islands by Si_0.8_Ge_0.2_ heteroepitaxy on rib-patterned Si (1 1 10) substrates in this work. Total energy density studies via finite-element methods (FEM) were also carried out for discussion on the possible evolution routes on the patterned substrate and their underlying driven forces.

## Methods

9 × 9 mm^2^ pieces of Si (1 1 10) substrates were patterned with a Leo Supra 35 field emission scanning electron microscope (FE-SEM) and subsequent reactive ion etching in an Oxford Plasmalab 80 reactor into 200 × 200 μm^2^ large arrays of parallel ribs along the [1 −1 0] direction. And the periodicities range from 0.5 to 2.5 μm, with the bottom width of the ribs ranging from 0.4 to 2.4 μm. The samples were chemically pre-cleaned using the RCA method. Immediately after a final hydrofluoric acid treatment, the samples were loaded into Riber® SIVA45 system for the molecular beam epitaxy (MBE) growth. After an in situ thermal desorption step at 950 °C for 10 min, a 30-nm-thick Si buffer layer was deposited while ramping the substrate temperature from 450 to 520 °C. Subsequently, 150 monolayers (ML) of Si_0.8_Ge_0.2_ were deposited at 650 °C with a rate of 0.12 Å/s. This thickness has been selected based on the successful formation of SiGe nanowires on both singular Si (1 1 10) [19] and patterned Si (0 0 1) [26] substrates without introduction of defects. As in previous work, the surface morphologies were characterized via ex situ atomic force microscopy (AFM) using a Veeco® Dimension 3100 system in the tapping mode. The AFM images were processed with the Gwyddion® analysis package. The experimental geometry was used for finite-element method (FEM) calculations of the total energy density. Modeling based on the experimentally observed geometry was performed by a FEM analysis of the Ge nanowires with the commercial COMSOL Multiphysics® 4.3 package.

## Results and Discussion

**Fig. 1:**
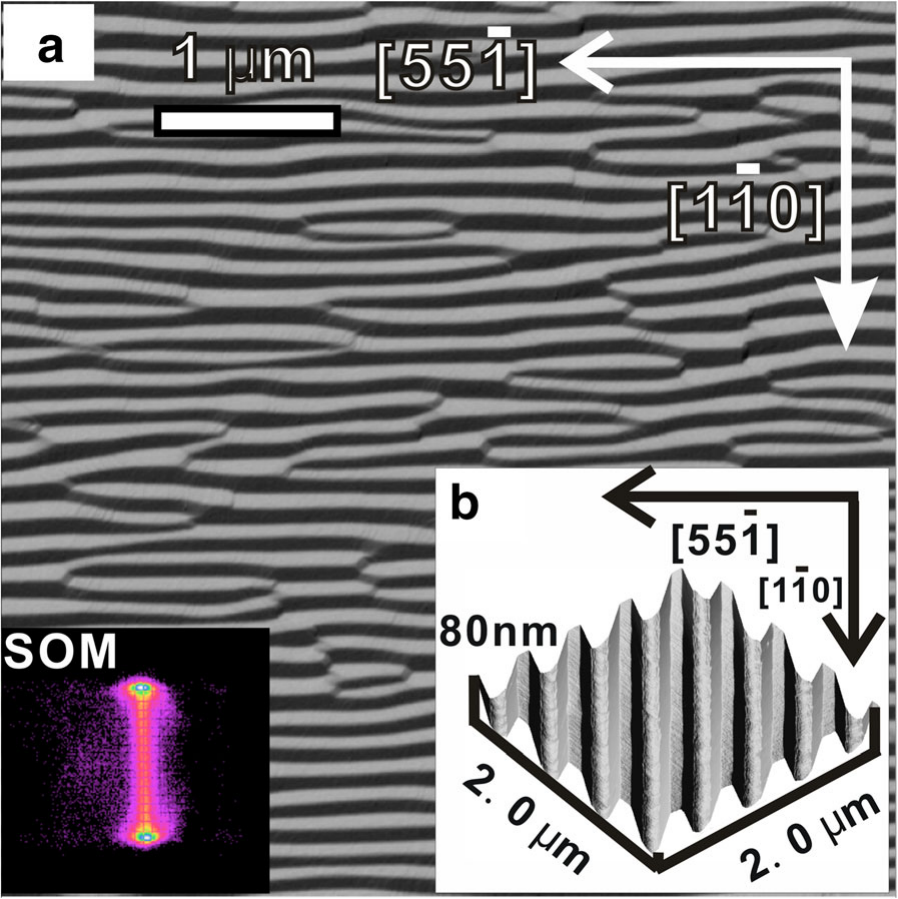
**a** A 3D rendering of AFM image for the rib-patterned Si (1 1 10) template with the ribs aligned along [1 −1 0]. **b** A derivative AFM image for the reference sample of an unpatterned Si (1 1 10) substrate after the growth of 150 ML Si_0.8_Ge_0.2_ at 600 °C

Figure 1a shows the derivative view of a 5 × 5 μm^2^ AFM image for a reference sample after the growth of 150 ML Si_0.8_Ge_0.2_ on a flat Si (1 1 10) substrate at 650 °C. The {105} faceted ripples dominated the whole surface, as can be seen in the surface orientation map (SOM; lower left inset). They are oriented along the [5 5 −1] direction, with the average ripple width of 140 ± 20 nm via 2D Fourier transform analysis (not shown here). The surface morphology of a typical rib-patterned Si (1 1 10) template used in this work is shown in Fig. 1b with a three dimensional (3D) rendering of the AFM image for an area of 2 × 2 μm^2^ and with the ribs’ orientation aligned along [1 −1 0] as a lower right inset in Fig. 1a. The periodicity of the ribs is about 500 nm, and the top width of the ribs is about 200 nm.

**Fig. 2:**
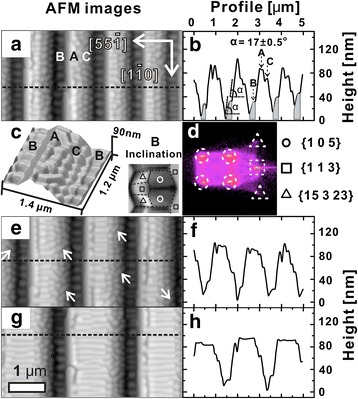
**a**, **c**, **e** 5 × 2.5 μm^2^ AFM images for the rib-patterned Si (1 1 10) templates with the top width of 450, 800, and 1100 nm after growth of 150 ML Si_0.8_Ge_0.2_ at 600 °C. **b**, **d**, **f** The cross-sectional profiles measured along the corresponding *dark guiding lines* shown in (**a**), (**c**), and (**e**)

Figure 2a, c are the top view and the 3D rendering of a 5 × 2.5 μm^2^ AFM image for the patterned Si (1 1 10) templates with the rib-top width of 450 nm after growth of 150 ML Si_0.8_Ge_0.2_ at 650 °C. And Fig. 2b is the cross-sectional profiles measured along the dashed guiding lines in Fig. 2a. Figure 2d is the SOM which helps to determine the faceting of the surface morphology. The AFM images show the top of the ribs has already been dominated by the {105} facet-bounded SiGe nanowires labeled as “A” with the width of about 200 nm as confirmed by SOM in Fig. 2d. The ribs are connecting two neighboring sidewalls, one of which is fully decorated with SiGe pyramid-shaped islands (labeled as “C” in Fig. 2a–c) featured with {105} facets as well according to the SOM. The symmetric geometry of the pyramids indicates that the sidewall they are sitting on has been faceted into an (0 0 1) surface. The opposites sidewall of the ribs are rather smooth with an inclination angle *α* = 17 ± 0.5° measured from the profile in Fig. 2b, which can be attributed to the {113} facets by SOM in Fig. 2d as well. This sidewall connects to the valley between the neighboring ribs, where short nanowires have also formed and have been labeled as “B” with an average width of about 200 nm. Nevertheless, these nanowires are terminated with dome-like {15 3 23} facets and {1 1 3} facets as indicated by the inclination map of two nanowires in the inset of Fig. 2c, and the SOM in Fig. 2d confirms these observations. With the bottom width increased up to 1.5 μm, the morphology of the rib top develops into the {105} facet-bounded nanowires as well, as shown in Fig. 2e, f. The length of these nanowires are limited by the width of the rib top, which is about 200 ± 50 nm as indicated in Fig. 2f. Besides the islands, short huts along the [100] direction can also be observed as pointed with white arrows in Fig. 2e. With the bottom width of the rib increasing up to 1.6 ± 0.1 μm, the rib top is fully covered by nanowires with the length of about 1.1 ± 0.1 μm as can be seen in Fig. 2g, h.

**Fig. 3:**
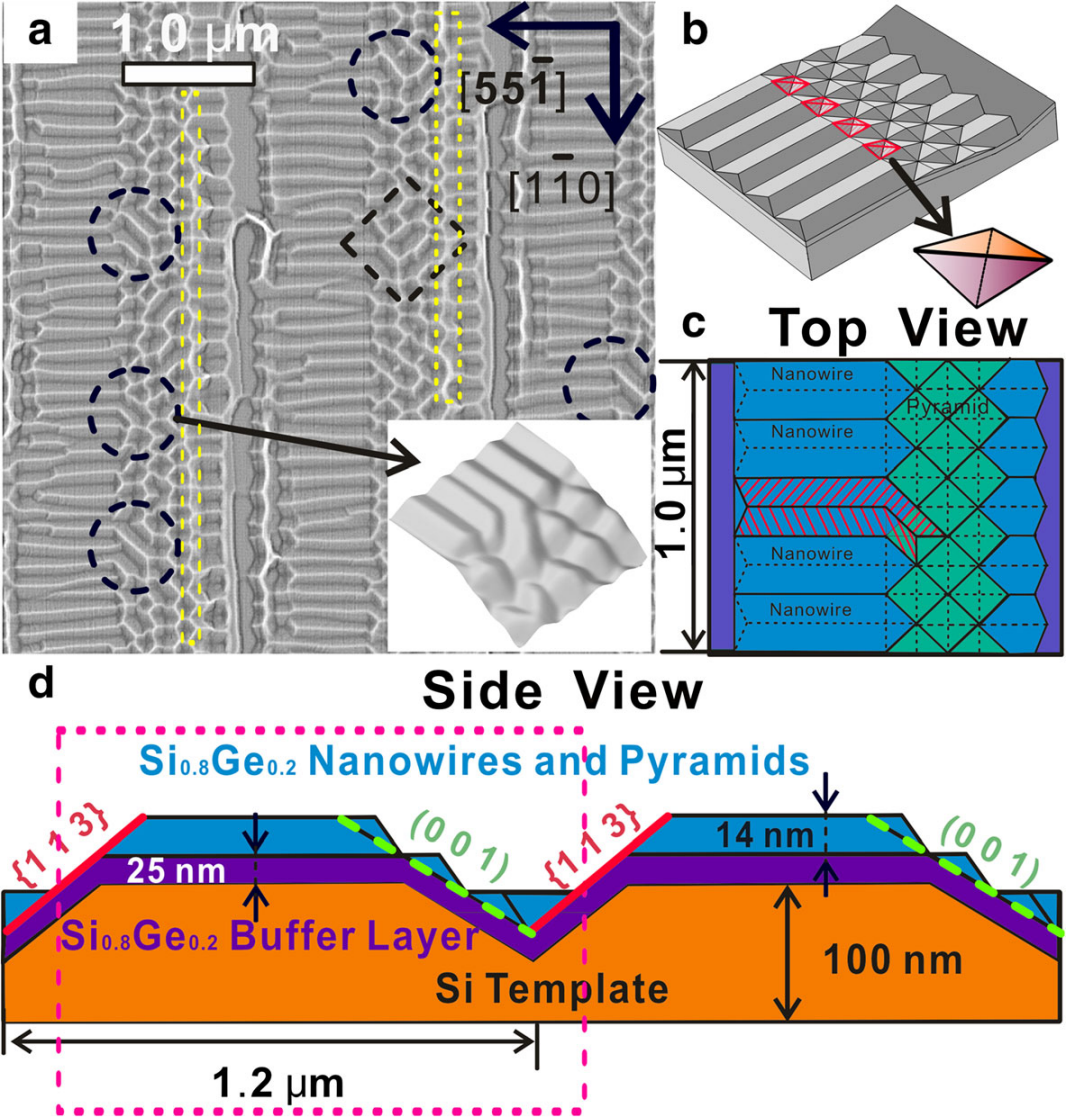
**a** A Laplacian-filtered AFM image for the sample with the rib bottom width of about 1.6 μm. *Inset*: 3D rendering of AFM image. **b** A 3D schematic for idealized sample morphology. **c**, **d** The top view and side view of the schematic

Figure 3a shows a Laplacian-filtered AFM image for the sample with the rib bottom width of about 1.6 μm to highlight the surface morphology, especially the faceting. It is well resolved that most of the nanowires on the rib top are terminated with two intersecting {105} facets [27]. Moreover, a self-connecting phenomenon at the rib shoulder between the nanowires on rib-top and the pyramids on the (0 0 1) faceted sidewall has been observed as highlighted with dashed circles in Fig. 3a. A zoom-in view of the AFM image in the down-right inset of Fig. 3a reveals that self-connecting is achieved through the filling of the vacancies between the neighboring nanowires and the pyramids with ad-atoms, which enables the merging of the two opposite {105} facets of the nanowires seamlessly to form extended nanowires on both the rib-top and the (0 0 1) faceted sidewall. Figure 3b is a 3D schematic for an idealized sample morphology with the whole Si (001) facet fully packed by pyramids, which is also the base for further finite-element simulation study. The diagonal of the pyramids is the same as the width of the nanowires on the rib-top of 200 nm, based on the AFM measurement. To form the extended nanowires, a tetrahedron section highlighted with red lines between for the nanowire and the pyramid is to be filled as shown in Fig. 3b. Figure 3c is the top view of the schematic where the two smoothly self-connected {105} facets of extended nanowires are marked with shadows to indicate the final surface morphology. While the coexistence of SiGe huts with the pyramids on both flat Si (001) and Si (1 1 10) substrates has been observed and studied systematically [26, 27], the self-connecting of the nanowires with the pyramids at the rib shoulder between the (1 1 10) rib-top and the (0 0 1) faceted sidewall has not been reported yet. Many huts can be observed sitting on the faceted sidewalls at various sites in the AFM images as highlighted by square dashes shown in Fig. 3a, although they are missing in Fig. 3b to simplify the model based on the observation that they are not observable overall, as Fig. 3a suggests. Meanwhile, we also find the absence of the self-connecting phenomenon at the bottom of the sidewall connecting to the {1 1 3} facet as highlighted by the yellow dashed rectangle in Fig. 3a.

The profile of the model with the information on the faceting and concentration of the materials is shown in Fig. 3d. The model itself is constructed based on the AFM results of the sample shown in Fig. 3a. A 100-nm-thick Si is chosen as the template. The rib is 56 nm in height, with a 25-nm-thick Si_0.8_Ge_0.2_ wetting layer shell painted in violet in Fig. 3d and a Si substrate core. The width of the Si_0.8_Ge_0.2_ nanowires is 200 nm, and thus, they are 14 nm in height, based on the AFM measurement as well. The width of the pyramids is fixed as the same value of the nanowires, i.e., 200 nm. The position and the orientation of the {113} faceted sidewall and the pyramid-covered (0 0 1) sidewall are also highlighted with a red solid line and green dashed line, respectively.

In this work, the rib-patterned template provides geometric condition to achieve the coexistence of both (1 1 10) faceted rib top and the (0 0 1) faceted sidewall. This leads to the simultaneous formation of nanowires on the rib top and the packed pyramids on the sidewall. Moreover, the onset of the self-connections between the nanowires and the pyramids at the rib shoulder and between the pyramids on the sidewall has also been observed. These results raise the question whether the self-connecting occurs at any preferential site, e.g., at the rib shoulder or at a rather random site without preference. The other question is: what is the driving force for the self-connecting of the nanowire-pyramid and pyramid-pyramid pairs? That is, is there any other energetically favorite way to distribute the volume of the tetrahedron with the concentration of the deposited materials being fixed. To answer these two questions, total energy density study for the deposited system including the nanowires and the pyramids using FEM calculations were carried out. The total energy density *ρ*_tot_ is given by *ρ*_tot_ = (*E*_str_ + *E*_surf_ + *E*_edge_) / *V*_tot_, where *E*_str_, *E*_surf_, and *E*_edge_ stand for the elastic strain energy, surface energy, and edge energy terms, respectively [17]. *V*_tot_ is the total volume for the total deposited materials in the models including the nanowires, huts, and the pyramids, which has a fixed value in this work. The elastic strain energy density *ρ*_str_ and the surface energy density *ρ*_surf_ are also defined by *ρ*_str_ = *E*_str_ / *V* and *ρ*_surf_ = *E*_surf_ / *S*, where *S* is the total surface area of the whole deposited geometry. While the {105} surface dominates the surface of the nanowires and the pyramids, the (0 0 1) facets and {1 1 3} faceted sidewall are also included in our following models. The surface energy density of Ge (001) is about 6.1 eV/nm^2^ [37]. As for the {1 1 3} facets, the key parameters of Ge {1 1 3} surface energies are adopted from the recent progress in ab initio calculation [38]. It should be noted that although the strain energy calculations are done with the Ge concentration being set to 20%, surface parameters for pure Ge {1 0 5}, (0 0 1), and {1 1 3} facets are adopted due to the dominating Ge surface segregation observed in previous studies [9, 14, 39]. This means that the trend of the surface energy with strain is implemented in the model and the effective value of the surface energy is computed by taking into account the real strain on these facets. Finally, the edge energy term *E*_edge_ is given by *E*_edge_ = *ρ*_edge_ × *L*, where *ρ*_edge_ is the energy density of the top and two basal ripple facet intersections, and *L* is the length of the nanowire. In our calculation, *ρ*_edge_ = 3.7 eV/nm is adopted for the {105}/{105} edge as well as the proposed (1 1 10)/{1 0 5} and (0 0 1)/{1 0 5} edges [17]. During the calculation, periodic boundary conditions are applied to both [5 5 −1] and [1 −1 0] directions. Thus, the region in the pink dashed line in Fig. 3d is used to perform the FEM simulation.

**Fig. 4:**
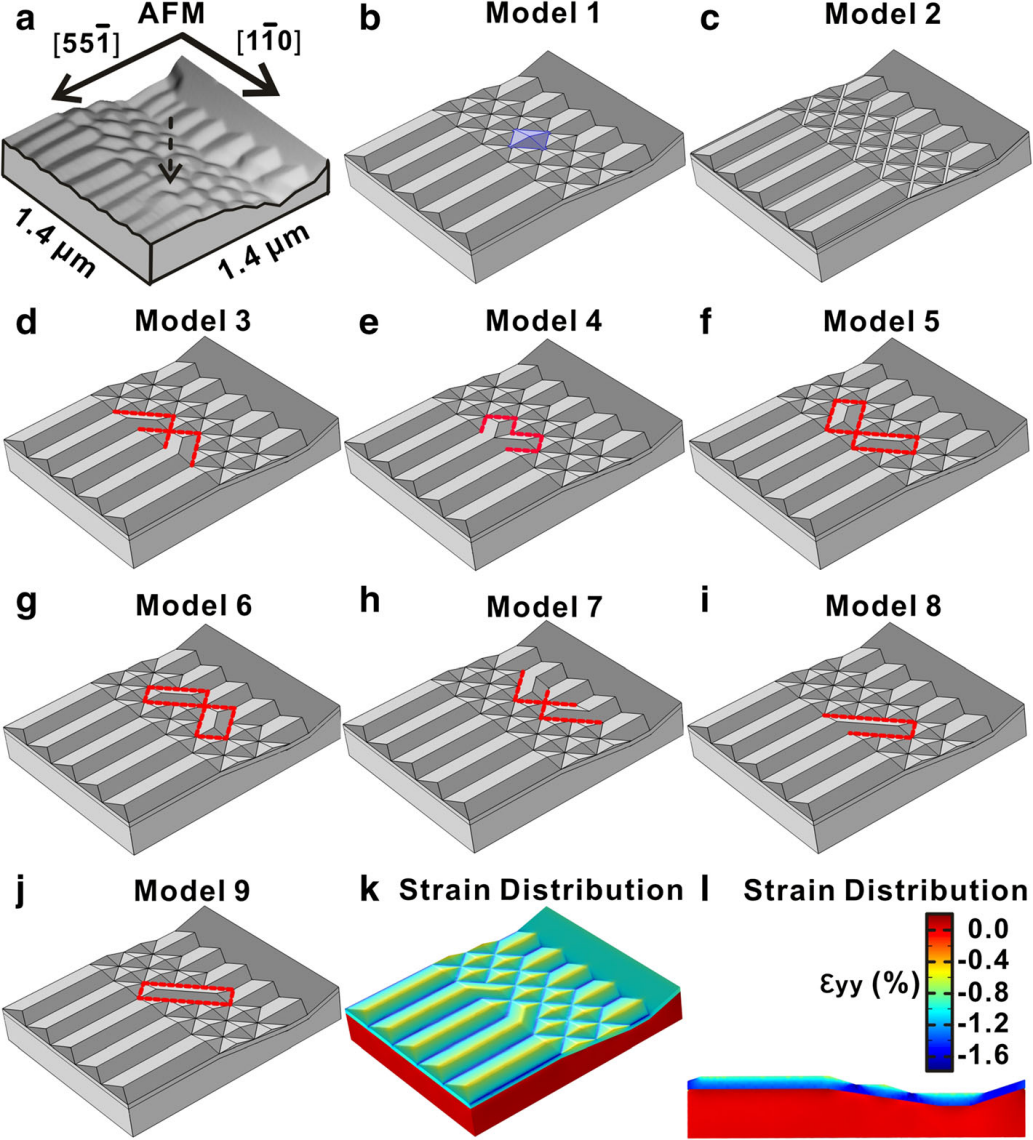
**a** A 3D rendering of AFM image for the rib-patterned Si (1 1 10) template with the ribs aligned along [1 −1 0]. **b**–**j** The different models used in FEM calculation for total energy density comparison. **k**, **l** The 3D view and cross-sectional view for the calculated strain energy distribution along y-axis in model #3

As an indication for the experimental origin of the proposed models, Fig. 4a is a section of the rotated 3D rendering of the AFM image in Fig. 3a. The black arrow indicates a self-connecting between the nanowire and the pyramid at the rib shoulder. Figure 4b–j shows the final improved models for the calculation with the self-connecting effect on the sidewall being taken into consideration. The major strategy is to compare the total energy density of the various models created by the distribution of the fixed amount of Si_0.8_Ge_0.2_ materials onto the basic model dominated by the combination of nanowires and pyramids on the rib-patterned template as shown in Fig. 3b with different configurations. The volume of the distributed Si_0.8_Ge_0.2_ materials is equal to the volume of two tetrahedron sections. Figure 4b, c shows models #1 and #2 representing the two alternative configurations, respectively. In Fig. 4b, the distributed ad-atoms accumulate at the valleys in between the adjacent nanowires and the pyramids to fill the bottom of the concave valleys as a kinetic-driven behavior [40]. In Fig. 4c, the size of a single specific pyramid is increased with the accumulation of the additional SiGe materials as highlighted with blue. The rest of the models, i.e., models #3, #4, #5, #6, #7, #8, and #9 with different configurations of the tetrahedrons connecting the pyramids, are proposed partly based on the experimental observations and are shown in Fig. 4d–j, respectively. Indeed, model #7 shown in Fig. 4h has not been observed in AFM images. And we also input this model into the calculation to investigate the reason for the absence of this configuration. In all these models, two tetrahedron sections are inserted to fill the vacancies between the adjacent nanowire/pyramid or pyramid/pyramid pairs to simulate the self-connecting phenomenon. In Fig. 4d, two huts are formed with two separate nanowire/pyramid pairs. In Fig. 4e, the situation that one nanowire is connecting to two separate pyramids simultaneously is considered. Models in Fig. 4f, g are used to simulate the cases that the self-connecting occurs between the neighboring pyramids at different sites on the Si (001) faceted sidewall. Then in Fig. 4h, the two tetrahedron sections are inserted to form the two separate huts connecting the nanowires sitting at the bottom of the rib valley and the pyramids at the sidewall. Elongated self-connecting is also taken into consideration with the models in Fig. 4i, j by aligning the two tetrahedron sections in a line. With these models, the typical configuration for the self-connecting observed are all represented. The 3D view and the cross-sectional view for the calculated strain energy distribution for the nanowires and pyramids in model #3 are shown in Fig. 4k, l.

All the above models are then input into FEM simulation. The calculated results are exhibited in Table 1. The total volume *V*_tot_ for the nanowires and the pyramids for all these models is determined to be 6.2632 × 10^6^ nm^3^. The total surface area *S*_tot_ for model #1 is 1.061 × 10^6^ nm^2^, and for model #2 1.049 × 10^6^ nm^2^. For model #1, the size expansion of the center pyramid leads to the increasing of the exposed area. For model #2, the flattening of the bottom of the valleys with (0 0 1) and (1 1 10) facets for the pyramids and nanowires cause the reduction of the total exposed area. The remaining seven models have the same value of the surface area of 1.051 × 10^6^ nm^2^ due to the fact that they share similar geometry with different sites for the inserted tetrahedrons. The total edge length *L*_tot_ of model #1 is slightly larger than the rest of the models due to the increase of the volume of the center pyramid by about 5%. On the other hand, the flattening of the valley bottom leads to the introduction of additional edges, thus significantly increasing the total edge length by 42%. Further results show that the calculated strain energy density *ρ*_str_ and the surface energy density *ρ*_surf_ for all the models are almost of the same value 0.064 ± 0.002 eV nm^−3^ and 6.325 ± 0.002 eV nm^−2^, respectively. So the calculated sum of the surface energy and the strain energy *E*_str_ *+ E*_surf_ for model #1 is about 7.109 × 10^6^ eV, which is about 0.9% larger than all the rest of the models. Then, we take the edge energy into consideration. As can be seen in Table 1, the edge energy for model #2 is 9.843 × 10^4^ eV, which is about 35~40% higher than all the other models. This is due to the fact that in model #2, additional (1 1 10)/{1 0 5} and (0 0 1)/{1 0 5} edges are introduced during the flattening of the valley bottom. And in the other models, the filling of the vacancies with tetrahedrons can replace the four pyramid edges with one top edge, which effectively reduces the edge energy for the whole system.

**Table 1:** The calculated results with FEM for the nine proposed models with different configurations of surface morphology

	Simulation model serials								
	#1	#2	#3	#4	#5	#6	#7	#8	#9
*V*_tot_ (×10^6^ nm^3^)	6.263								
*S*_tot_ (×10^6^ nm^2^)	1.061	1.049	1.051						
*L*_tot_ (×10^6^ nm)	0.020	0.027	0.019						
*E*_str_ (×10^5^ eV)	4.016	4.100	4.014	4.013	4.010	4.009	4.016	4.014	4.011
*ρ*_str_ (eV nm^−3^)	0.064	0.066	0.064	0.064	0.064	0.064	0.064	0.064	0.064
*E*_surf_ (×10^6^ eV)	6.707	6.635	6.645	6.645	6.645	6.645	6.645	6.645	6.645
*ρ*_surf_ (eV nm^−2^)	6.324	6.327	6.325	6.325	6.325	6.325	6.325	6.325	6.325
*E*_str_ *+ E*_surf_ (×10^6^ eV)	7.109	7.045	7.046	7.046	7.046	7.046	7.046	7.046	7.046
*E*_edge_ (×10^4^ eV)	7.299	9.843	7.047	7.122	7.047	7.047	7.159	7.047	7.047
*E*_tot_ (×10^6^ eV)	7.182	7.143	7.117	7.117	7.117	7.117	7.118	7.117	7.117
*ρ*_tot_ (eV nm^−3^)	1.147	1.141	1.136	1.136	1.136	1.136	1.137	1.136	1.136

Thus, the total energy and the total energy density can be obtained. These results show that the total energy density for both models #1 and #2 are 1.147 and 1.141, which are about 1 and 0.4% higher than the rest models, respectively. For model #1, the higher surface area, and thus the higher surface energy, contributes to the higher total energy. This phenomenon has been observed before during the synchrotron radiation-based grazing incidence small angle X-ray scattering measurement [41], in which the stabilization of the prism structure is attributed to the strain-dependence of the {105} surface energy. And our FEM results seem to support this in terms of total energy density analysis. As for model #2, although the edge energy only takes up about 1% of the total energy, the much higher edge energy still plays a key role in the comparison of total energy as discussed before [17], which rules out the possibility to form the enlarged pyramids on the patterned substrate. The total energy density difference exhibited above is quite small. Nevertheless, this is not uncommon that a small total energy difference in the order of 1% may lead to the onset of the energetically preferential geometry [19, 27], so long as the *quasi* equilibrium growth condition is fulfilled. The rather low deposition rate of 0.12 Å/s and medium temperature of 650 °C during our MBE growth exactly provide the favorite condition which enabled the thermodynamics the dominating driven force for the observed phenomena.

The rest of the configurations all behold almost identical total energy density of 1.136 eV/nm^3^ no matter where the inserted tetrahedrons are, with the exception of model #7 that has the value of 1.137 eV/nm^3^. This result fits the fact that all the above configurations, except #7 with only about 0.88‰ larger in the total energy density, have been experimentally observed. It is again the difference in the edge energy that counts for these results. The same value of the total energy density for configurations #3, #4, #5, #6, #8, and #9 indicates the self-connecting can onset at any vacancies to form hut-like structures without energetically preferential site, once the (0 0 1) faceting is achieved during the SiGe epitaxy [42].

FEM results provide possible explanations for the experimentally observed phenomena in terms of energetic comparison. The driving force for the onset of the self-connecting instead of all competing configurations is attributed to the minimization of the total energy density. And all the components of the total energy, including the minimal edge energy term, contribute to the determination of the final morphology.

It should be noted that the formation of the nanowires on (1 1 10) faceted rib top and the pyramids and the elongated huts on (0 0 1) faceted sidewall are mainly two separate processes. Early work shows the formation of pyramids on Si (001) substrate after deposition of 3.8 nm Si_0.7_Ge_0.3_ [43]. With similar coverage of 30 ML, Si_0.8_Ge_0.2_ nanowires formation also onsets on Si (1 1 10) substrate [41]. Thus, due to the rather low growth rate and medium growth temperature used in our work, it can be expected that the nanowires and the pyramids form simultaneously on both facets. Further geometry evolution for the Si-rich SiGe on flat Si(1 1 10) substrate has also been elaborated in previous work [27, 41]. But in this work, the rib-patterning along the [1 −1 0] direction induces the truncating for nanowires at the rib shoulders. Therefore, we observed that most of the nanowires on the rib top are terminated with two intersecting {105} facets, as shown and discussed in Fig. 3a, which coincides with the “tadpole” configuration onsets during the geometric evolution from 1D nanowire towards 3D island [27]. Since this configuration were evolved from the (0 0 1) terminated ripple, it is reasonable to suppose the nanowire ends terminated with intersecting {105} facets on the rib shoulder also developed from (0 0 1) faceted termination during the growth. On the other hand, ab initio calculations show the potential fluctuations on reconstructed Ge {105} surface are very small [44], and the surface migration of SiGe ad-particles on it is fast and almost isotropic, which can significantly enhance the interaction between the nanowires on the rib top and the pyramids on the (0 0 1) sidewall. Indeed, the observed width of the nanowires and length of pyramid diagonal finally evolved into the same value of about 200 nm. Thus, at the shoulder sites, the nanowires and the pyramids generally self-arranged into the close packing configuration, with each nanowire wedging in between two neighboring pyramids, as observed in Fig. 3a and schematically illustrated in Fig. 3b, c. The well-developed geometry at the rib shoulder paves the evolution route for further connecting between the nanowires on (1 1 10) faceted rib top and the pyramids/elongated huts on (0 0 1) faceted sidewall.

**Fig. 5:**
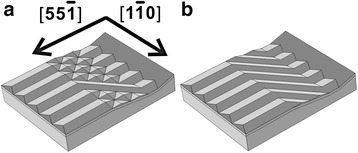
**a**, **b** The schematic models for one single long nanowire (**a**) and a complete nanowire array (**b**) sitting on both the connected (1 1 10) faceted rib top and (0 0 1) faceted sidewall

On the other hand, the formation the elongated huts through the self-connecting between the pyramids is indeed the precursor before the final formation of the Si-rich SiGe nanowires on Si (0 0 1) substrate [26, 42, 45]. Once the self-connecting can be achieved at the shoulder site, the long SiGe nanowire with random direction along orthogonal [0 −1 0] directions covering both the rib top and the sidewall as shown in Fig. 5a can be expected with increasing coverage and moderate annealing. However, to achieve a completely aligned nanowire array fully covering both connected facets as shown in Fig. 5b, alternative growth method with well-tuned growth condition [10] based on the thermodynamic wave model [17] and the additional geometric restrictions [26] have to be applied.

## Conclusions

In conclusion, heteroepitaxy of Si_0.8_Ge_0.2_ onto rib-patterned Si (1 1 10) templates oriented along [1 −1 0] leads to the formation of SiGe nanowires on the rib top along [5 5 −1] direction, as well as the sidewalls bound with respective (0 0 1) and (1 1 3) facets. While the (1 1 3) dominated sidewalls keep smooth, the (0 0 1) faceted sidewalls are fully decorated with closely packed SiGe pyramids and huts. Finite-element simulations reveal that the formation of the huts on Si (001) via the self-connecting of the adjacent pyramids, as well as between the nanowires and the pyramids at the rib shoulder sites are both driven by the minimization of the total energy density, while the competing geometric evolution routes via pyramid expansion and valley flattening are suppressed. These results provide a convenient solution to fabricate the self-assembled in-plane nanowires covering multi-faceted surfaces on the patterned templates.

## Abbreviations

3D: Three dimension
AFM: Atomic force microscopy
FEM: Finite-element methods
FE-SEM: Field emission scanning electron microscope
MBE: Molecular beam epitaxy
ML: Monolayer
SOM: Surface orientation map
VLS: Vapor-liquid-solid
